# Predictor Factors in the Success of Slow-Release Dinoprostone Used for Cervical Ripening in Pregnancies with Premature Rupture of Membranes

**DOI:** 10.5152/eurasianjmed.2022.21237

**Published:** 2022-02-01

**Authors:** Mehmet Obut, Gulşah Aynaoğlu Yıldız, Muhammed Hanifi Bademkıran, Süleyman Cemil Oğlak, Özge Yücel Çelik, Fatma Ölmez

**Affiliations:** 1 Department of Gynaecology and Obstetrics, Health Sciences University, Diyarbakır Gazi Yaşargil Training and Research Hospital, Diyarbakır, Turkey; 2 Department of Perinatalogy, Health Sciences University, Etlik Zübeyde Hanım Woman’s Health Training and Research Hospital, Diyarbakır, Turkey; 3 Department of Gynaecology and Obstetrica, Kanuni Sultan Süleyman Training and Research Hospital, Diyarbakır, Turkey

**Keywords:** Delivery, labor induction, prediction, premature rupture of membranes, prostaglandin E2

## Abstract

**Objective:** The study aimed to evaluate the factors affecting successful vaginal delivery in induction with slow-release dinoprostone at term pregnancy with premature rupture of membranes.

**Materials and Methods:** Pregnancies between 37^0/7^ and 41^6/7^ gestation weeks with premature rupture of membranes in which slow-release dinoprostone was used for cervical ripening were sought for inclusion in the study. Pregnancies with previous uterine surgery, multiple fetal gestations, chorioamnionitis, non-cephalic presentation, fetal distress at the time of admission, HIV positivity, and estimated fetal weight >4500 on ultrasonographic evaluation were excluded. The primary outcome of measures were factors affecting the success of vaginal delivery including maternal age, gestational weeks at delivery, initial Bishop score, parity, induction time, and induction–delivery time interval. To reduce the risk of overfitting in the study, penalized maximum likelihood estimation was performed instead of traditional logistic regression in the statistical analysis.

**Results:** A total of 1266 participants who met the study criteria were included in the study. Among the parameters evaluated for the prediction of successful vaginal delivery in cases with premature rupture of membranes, maternal age (*P* < .001), Bishop score (*P* < .001), parity (*P* = .01), induction time (*P* < .001), and induction–delivery time interval (*P* < .001) had an impact on success. The mean gestational week of the participants who had cesarean deliveries was lower than in those who had vaginal deliveries (*P* = .03); however, this was not a predictor factor of penalized maximum likelihood estimation (*P* = .70).

**Conclusion:** Basic parameters such as maternal age, induction time, parity, and Bishop score can be used to predict successful vaginal birth following dinoprostone slow-release vaginal insert administration.

**Main Points**

- Slow**-**release PGE2 vaginal insert is safe and effective in labor induction of cases with premature rupture of membranes (PROMs).
- The higher Bishop score and lower induction time in cases having PROM increases the probability of vaginal delivery.
- The predictor factors may help to predict successful vaginal delivery in cases with PROM.

## Introduction

Premature rupture of membranes (PROM) is defined as the loss of membrane integrity before labor begins and complicates approximately 5-8% of all pregnancies.^1,2^ The diagnosis is made by the presence of amniotic fluid flow from the cervical canal in a speculum examination or by performing tests on fluid taken from the vagina. Studies that compared labor induction with the expectant approach have shown that early induction reduces the rate of infectious morbidity (endometritis and chorioamnionitis) in the mother and the infant’s risk of early neonatal sepsis without adding additional fetal and maternal complications.^1-5^

There are two main methods for labor induction, mechanical (Foley catheter, Cook cervical ripening balloon, and osmotic dilatators) and pharmacologic (prostaglandin analogs and oxytocin). There has been some concern regarding infectious morbidity because the devices used in the mechanical method come into direct contact with the amniotic cavity.^4,5^ Also, there has been some concern regarding the efficacy of pharmacologic methods because of the possibility of the dissolved part of the drug being washed away by the leakage of amniotic fluid from the cervix. The most used pharmacologic methods for labor induction are oxytocin and prostaglandin E2 (PGE2). Studies that compared oxytocin with PGE2 were in favor of PGE2, especially in cases of unripening cervix.^6-9^ Cervical ripening with prostaglandins is thought to be closer to the natural cervical ripening mechanism. There are 3 forms of PGE2: gel, tablet, and the 10-mg controlled-release insert form. Prostaglandin E2 has been approved for cervical ripening and induction in many countries.^10,11^ Knowing factors that affect the success of delivery in cases with PROM can contribute to the decision-making process, quality of perinatal care, and may even be life-saving for the fetus.^12^ In this study, we aimed to investigate the factors that affected vaginal delivery success in pregnancies with PROM that underwent vaginal PGE2 insertion for the induction of labor.

## Materials and Methods

### Study Design and Participant Characteristics

After ethical consent was obtained from the Ethics Committee of Health Sciences University, Diyarbakır Gazi Yaşargil Training and Research Hospital Training and Research Hospital (Approval number: February 02, 2018/21), 25 678 pregnant women who gave birth between March 15, 2018, and March 14, 2019, were retrospectively reviewed from the hospital records. A total of 1266 pregnancies that met the study criteria were included in the study. To eliminate the possible effects of pre-term pregnancy and post-term pregnancy on the study results, pregnancies with PROM between 37 0/7 to 41 6/7 weeks of gestation were included in the study. To optimize the results, pregnancies with previous uterine surgery, multifetal gestations, breech presentation, HIV positivity, active genital herpes simplex virus findings, nonreactive non-stress test at admission, pregnancies rejecting vaginal birth, pregnancies with placenta previa, infection, or chorioamnionitis findings were excluded from the study.

The demographic, obstetric, and physical characteristics of the subjects, pregnancy-related complications, cervical examination findings during admission, gestational week, fetal sex, fetal birth weight, early neonatal outcomes, induction time, number of vaginal PGE2 inserts, and cesarean indications were evaluated. Induction of labor was considered successful in subjects who had vaginal delivery after a single or second vaginal PGE2 insert. Pregnancies that could not achieve vaginal maturation with a vaginal PGE2 insert and had to undergo cesarean section for any reason were considered unsuccessful.

### Procedure

Pregnancies with PROM with a Bishop score of six or less received a 10-mg controlled slow-release vaginal PGE2 insert, which released dinoprostone for up to 12 hours at a rate of 10 mg, 3 mg/h (Propess®, Ferring Pharmaceuticals, Germany), in the posterior fornix for cervical maturation according to our clinical protocol. We perform vaginal PGE2 insertion at the time of admission. Once the vaginal PGE2 insert has been placed, we waited until cervical ripening is achieved (Bishop >6) or until 12 hours after inserting the pessary. If cervical maturation cannot be achieved with the first vaginal PGE2 insert, a second insert is placed. If cervical ripening is not achieved within 24 hours after administering the first dose of PGE2, these pregnancies are delivered by cesarean section.

In the event of uterine tachysystole (more than 5 contractions per 10 minutes), or non-reassuring fetal heart rate monitoring, the PGE2 vaginal insert is removed immediately. Electronic fetal heart rate was monitored before performing the vaginal PGE2 insert and is monitored continuously thereafter.

Failed induction is defined as a Bishop score of <6 or a failure to enter active labor despite 2 vaginal PGE2 inserts being used and 24 hours elapsing since the first PGE2 insert.

Failure to progress was defined as cervical dilatation not increased in the first stage or the level of the fetal head not descending after 1 hour in the second stage of labor despite oxytocin augmentation or adequate uterine contraction in a 4-hour interval.

After cervical maturation, the pregnant woman is sent to the delivery room, and oxytocin augmentation is performed or followed spontaneously according to the contraction status.

## Statistical Analysis

Student’s *t*-test and the Mann–Whitney *U* test were used to compare continuous variables with and without normal distribution. Proportional data were compared using the chi-square test and Fisher’s test. Differences were considered to be significant when the *P* value was less than .05. This study used the penalized maximum likelihood estimation (PMLE) method to examine the relationship between the outcome and candidate predictors. The PMLE method was used instead of traditional logistic regression, especially in high-dimensional data because the variance was significantly reduced in the prediction of new subjects. Penalized maximum likelihood estimation maximizes the penalized log-likelihood instead of maximizing the log-likelihood made in traditional logistic regression. Bishop score, maternal age, induction time, and induction–delivery time intervals were identified as candidate predictors. Very low- or very high-frequency variables were not included in the model. As a result, 3 candidate variables were included in the final model. All statistical analyses were performed using the R software package, version 3.5.1 (R Statistical Software, Institute for Statistics and Mathematics, Vienna, Austria).

## Results

**Figure 1.**
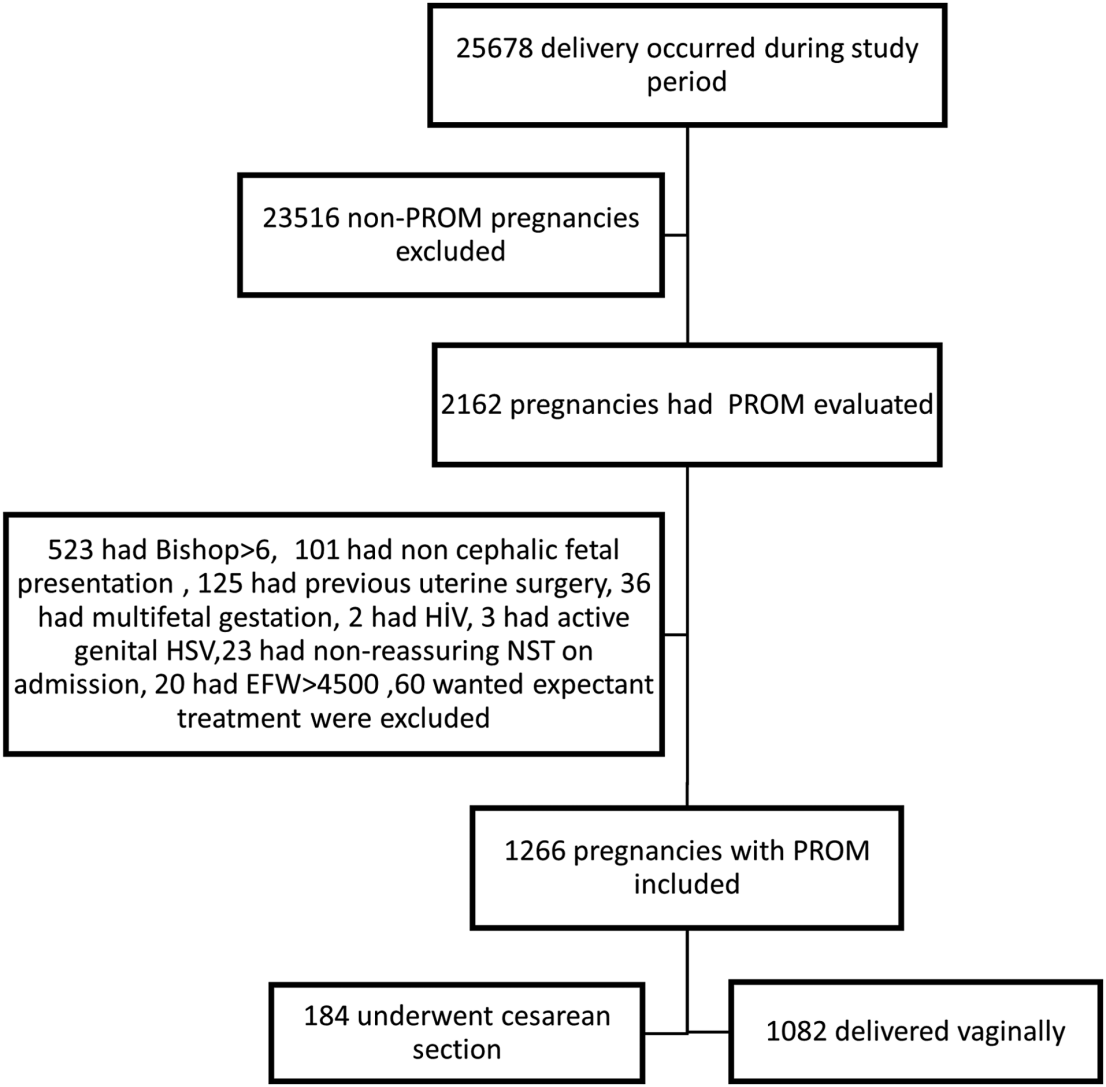
Description of the study follow-up.

A total of 25 678 deliveries occurred in our hospital during the study period, 1266 of which met the study criteria. A total of 1082 (86%) of these pregnancies were successful with slow-release vaginal PGE2 insert induction, and 184 (14%) were unsuccessful (Figure 1). The mean age of the participants was 24 years in the successful group and 26 years in the failed group. There was a statistically significant relationship between maternal age (*P* = .005) and gestational age (*P* = .03) and induction success, but no correlation was found between induction success and parity (*P* = .9). Also, women who required a second dose of Propess were less likely to have vaginal delivery compared with those who had only 1 dose (*P* < .001) (Table 1).

Table 2 shows the association of candidate predictors in the success of vaginal PGE2 inserts for vaginal delivery in women with PROM. Although increased maternal age (odds ratio (OR) = 0.94, 95% CI: [0.91-0.97]; *P* < .001), induction time (OR = 0.90, 95% CI: [0.87-0.93]; *P* < .001), and induction–delivery time interval (OR = 0.62, 95% CI: [0.41-0.78]; *P* < .001) decreased the possibility of vaginal delivery, Bishop score at induction (OR = 1.54, 95% CI: [1.23-1.94]; *P* < .001) and parity (OR = 1.18, 95% CI: [0.83-1.67]; *P* < .01) increased the possibility of vaginal delivery in pregnancies with PROM. There was no effect of gestational age at delivery and maternal disorder on the success of vaginal delivery in pregnancies with PROM.

The most common reasons for cesarean section were non-reassuring fetal heart rate (49%) and failure to progress (32%) (Table 3).

When the fetal results were compared, the fifth-minute APGAR scores were significantly lower in the cesarean section group (*P* < .001). The mean fetal weights were 3.140 g in the vaginal delivery group and 3.100 g in the failed group. This difference was statistically significant (*P* = .03). There was no difference between the groups in terms of fetal sex (*P* = .08) (Table 4).

## Discussion

The benefit of active labor management in PROM pregnancies has been confirmed by several studies.^1,2,13-15^ Especially in cases where membranes are not intact, hasten the delivery process is essential in preventing both maternal and fetal complications.^14,15^ The prolongation of labor is associated with an increased risk of maternal chorioamnionitis, postpartum fever, and neonatal infection.^16^ Also, prolonging the labor process increases participant dissatisfaction, as seen in questionnaires related to satisfaction with care during labor.^13,14^ In our clinic, we recommend active management in all PROM pregnancies and determine the active management model according to Bishop scores. We perform oxytocin augmentation in pregnancies with Bishop scores of 7 or more, and vaginal PGE2 inserts in pregnancies with Bishop scores of 6 or less. There are many publications in the literature evaluating the success of induction of vaginal PGE2 inserts in term pregnancies and factors affecting the success.^8,10-17^ However, the number of studies in PROM pregnancies is relatively small. Amniotic fluid flows into the vagina in PROM because of the loss of membrane integrity. Therefore, vaginal pH and vaginal microbiota change and also some dissolved PGE2 may be washed away. As a result, both the pharmacokinetics of vaginal dinoprostone and the subject’s response change.^17,18^ A study found that the induction time was shortened after vaginal lavage with saline before vaginal PGE2 inserts. The authors concluded that the reason for shortening induction time might change according to PGE2 bioavailability.^19^ In this study, the cesarean rate was 14%, showing that vaginal PGE2 inserts were safe and effective in PROM pregnancies.^19,20^

In our study, after vaginal PGE2 insert use in PROM pregnancies, we found that maternal age, Bishop scores, and duration of induction, and the induction to delivery time interval were factors that affected the success of prostaglandin E2 slow-release inserts in patients.

There are different results in the literature regarding the association between maternal age and induction success.^12,21^ Our study shows that older maternal age increased the rate of induction failure. The progression of maternal age increases the likelihood of obstructive factors such as obesity, hypertension, diabetes mellitus, and fetal macrosomia, and an increased parity number with increasing age decreases the likelihood of obstructive factors such as cephalo-pelvic disproportion. Therefore, we think that the effect of maternal age on birth success may vary between populations.

Bishop scores show cervical changes in response to labor and prostaglandins secreted due to changes in the cervix, affecting the success of induction in both PROM pregnancies and intact membrane pregnancies.^12,21-24^ They indicate that the cervix changes both anatomically when cervical changes occur and with various cytokines and biochemical substances secreted from the cervix, and these substances contribute to the progress of labor. However, the meta-analysis conducted by Kolkman et al^25^ found that the Bishop scores were frequently used despite the limited predictive capacity. Our study found that Bishop scores were one of the crucial independent variables that affected induction success.

Prolongation of delivery is associated with an increased risk of maternal chorioamnionitis, postpartum fever, and neonatal infection.^16,26^ It also increases patient dissatisfaction, as seen in questionnaire studies related to satisfaction with care during labor.^27^ In our study, the lower induction time and the induction to delivery time, the more successful was vaginal delivery. In other words, cesarean rates increased when the delivery process was prolonged, and nonprogressive action was the second most common cause of cesarean section. We think this was due to increased maternal and fetal complication risks and adverse effects on patient satisfaction. Prolongation of the delivery process also affects the association between parity and labor. We assumed that the reason for finding parity to be a statistically nonsignificant predictor in the success of labor induction in PROM pregnancies was the prolongation of this process.

The relationship between BMI and induction success continues to be investigated, and studies in PROM pregnancies suggest an inverse relationship between BMI and induction success.^22,28^ However, the pathophysiology of the adverse effects of obesity on labor is not fully understood. In the study of Zhang et al.^29^ the uteri of overweight women who underwent elective cesarean section contracted with less force and frequency. Garabedian et al^30^ reported that as BMI increased, oxytocin receptor expression increased, probably due to a decrease in receptor sensitivity. Contrary to these studies, Batinelli et al^26^ found no such relationship. In our study, in line with later, no correlation was found between BMI and induction success.

There are conflicting results in the literature regarding gestational week and induction success. Although Creane et al^31^ found an association between increased gestational age and decreased fetal weight and success of induction in a 2006 meta-analysis, Riboni et al^21^ found no significant relationship between gestational age and induction success. Park et al^24^ found that fetal macrosomia was an independent risk factor for induction failure in PROM pregnancies. In our clinic, when active labor management is selected for PROM pregnancies, estimated fetal weight is determined in ultrasonographic examinations, and cesarean section is recommended for fetuses with macrosomia. Therefore, in this study, we cannot comment on the success of Propess in the case of fetal macrosomia in women with PROM. The mean fetal weight was 3140 g in the vaginal delivery group and 3100 g in the cesarean delivery group, and this difference was statistically significant. Some studies are showing that the cesarean delivery rates are higher in small-for-gestational-age infants.^32^ However, in our study, we could not use fetal weight in the prediction of vaginal delivery because they were revealed only after the birth.

In 2013, Torricelli et al^33^ found that fetal sex affected induction success independent of parity and that male fetuses were more likely to be taken to cesarean section. Also, meconium staining rates, induction time, and birth weights of male fetuses were high. However, such a relationship was not found in fetal sex in a study conducted Ashwal et al.^34^ In our study, there was no relationship between fetal sex and induction success.

The strengths of this study are the high number of samples and the use of the same dinoprostone vaginal insert system as an induction method for all pregnancies in the study. To reduce the risk of overfitting in the study, PML was performed instead of traditional logistic regression in the statistical analysis. However, some limitations should be mentioned; first, we did not measure the cervical length in ultrasonography, which has been shown to have value in the prediction of successful vaginal delivery.^35^ Second, this study had a retrospective design. Finally, we did not evaluate the outcomes of subjects who underwent labor induction with methods other than dinoprostone.

In conclusion, dinoprostone is effective in labor induction in cases of PROM. Basic parameters such as maternal age, induction time, parity, and Bishop scores can be used to predict successful vaginal birth following dinoprostone slow-release vaginal insert administration.

## Figures and Tables

**Table 1.** Evaluation of Demographic Parameters Between the 2 Groups

Characteristic	Vaginal Delivery (n = 1082), 86%	Cesarean Section (n = 184), 14%	*P*
			
Maternal age (year), (median [interquartile range (IQR)])	24 (21-29.7)	26 (21-32)	**.005**
Body mass index (kg/m^2^) (median [IQR])	25 (19.3-30.5)	26 (19.6-31)	.295
Parity (median [IQR])	1 (1-2)	1 (1-2)	.9
Gestational age (weeks) (median [IQR])	39 (37-39)	38 (37-39)	**.03**
No of propess			
1	98 (1062)	82 (151)	<.001**
2	2 (20)	18 (33)

Values are presented as (n) %; median (interquartile range); bold values represent *P* < .05.

**Table 2.** Evaluation of Some Labor Characteristics of the 2 Groups

Variables	Odds Ratio, 95% CI	*P*
Maternal age	0.94 (0.91-0.97)	<.001
Gestational age at delivery	1.02 (.90-1.16)	.70
Bishop score at induction	1.54 (1.23-1.94)	<.001
Parity	1.18 (0.83-1.67)	**.01**
Induction time	0.90 (0.87-0.93)	<.001
Maternal disorder	0.65 (0.42-1.02)	.06
Dinoprostone amount	0.5 (0.2-1.15)	.1
İnduction-delivery time interval	0.62 (0.41-0.78)	<.001

Penalized adjusted odds ratios to track the association of candidate predictors with the success of dinoprostone vaginal delivery in women as rupture of membranes.

Values are presented as (n) %; median (interquartile range); bold values represent *P* < .05.

**Table 3.** Reason for cesarean section

Reason for cesarean section	(n) %
Non-reassuring fetal heart rate	49 (91)
Failure to progress	32 (60)
Induction failure	10 (20)
Aggravation of maternal state	7 (13)

**Table 4.** Evaluation of Neonatal Characteristics of the 2 Groups

Characteristic	Vaginal Delivery (n = 1082) 86%	Cesarean Section (n = 184) 14%	*P*
Apgar			<.001**
1-min APGAR score < 4, n (%)	1 (0.1%)	5 (2.7%)	
5-min APGAR score < 7, n (%)	4 (0.4%)	17 (9.3%)	
Fetal weight , g (median [interquartile range])	3.140 (2.900-3.400)	3.100 (2.750-3.350)	.03*
Fetal gender			.08
Male, n (%)	528 (48.7%)	102 (55.4%)	
Female, n (%)	556 (51.3%)	82 (44.6%)	

Values are presented as (n)%; median (interquartile).

*Bold values represent *P* < .05 and **bold values represent *P* < .001.
